# Key Predictors of Neonatal Respiratory Compromise in Placenta Accreta Spectrum: An 11-Year Retrospective Cohort Study

**DOI:** 10.3390/jcm15041542

**Published:** 2026-02-15

**Authors:** Praew Chareesri, Supaporn Dissaneevate, Anucha Thatrimontrichai, Gunlawadee Maneenil, Manapat Praditaukrit, Pattima Pakhathirathien, Savitree Pranpanus, Chanon Kongkamol

**Affiliations:** 1 Department of Pediatrics, Division of Neonatology, Faculty of Medicine, Prince of Songkla University, Songkhla 90110, Thailand; praew.c@psu.ac.th (P.C.); tanucha@medicine.psu.ac.th (A.T.); mgulawa@medicine.psu.ac.th (G.M.); manapat.p@psu.ac.th (M.P.); ppattima@medicine.psu.ac.th (P.P.); 2 Department of Obstetrics and Gynecology, Faculty of Medicine, Prince of Songkla University, Songkhla 90110, Thailand; savitree.p@psu.ac.th; 3 Department of Family Medicine and Preventive Medicine, Faculty of Medicine, Prince of Songkla University, Songkhla 90110, Thailand; kchanon@medicine.psu.ac.th

**Keywords:** clinical prediction rules, morbidity, neonatal intensive care unit, newborn, placenta accreta, placenta increta, placenta percreta

## Abstract

**Background/Objectives**: Placenta accreta spectrum (PAS) is associated with substantial maternal and perinatal morbidity and may lead to respiratory distress in newborns. However, limited evidence exists regarding predictors of respiratory compromise (RC) in neonates born to pregnancies complicated by PAS. **Methods:** This retrospective cohort study included neonates born to pregnancies complicated by PAS between 1 January 2014 and 31 December 2024. Independent predictors of RC were identified using logistic regression, and a weighted scoring model was developed. Model performance and internal validity were assessed using area under the receiver operating characteristic curve, calibration plots, and bootstrap re-sampling. **Results:** Among 237 neonates born to PAS-complicated pregnancies, 112 (47.3%) experienced RC. Six independent predictors were identified and assigned weighted points: maternal vaginal bleeding within 24 h before delivery (2 points); placenta type—accreta (reference), increta (1 point), and percreta (2 points); absence of antenatal corticosteroid use (1 point); gestational age—29–31 weeks (5 points) and 32–36 weeks (3 points); birth weight < 2500 g (2 points); and male sex (2 points). At a score threshold of 7, the model demonstrated good discrimination, with an area under the receiver operating characteristic curve of 0.75, sensitivity of 67.6%, and specificity of 72.9%. **Conclusions:** A predictive score > 7 provides fair discrimination for identifying RC in neonates born to pregnancies complicated by PAS and may assist clinicians in identifying high-risk infants who require closer monitoring and early respiratory support.

## 1. Introduction

Placenta accreta spectrum (PAS) is a pathological continuum of abnormal placental adherence and invasion into the uterine wall and is classified into three levels of severity: placenta accreta (attachment to the myometrium without invasion), placenta increta (invasion into the myometrium), and placenta percreta (penetration through the serosa and into adjacent pelvic organs, such as the bladder or bowel) [1,2]. The incidence of PAS has increased worldwide, affecting 1 in 533–731 pregnancies [3,4]. This increase is largely attributable to rising cesarean delivery rates and other uterine interventions, such as curettage and myomectomy. Each additional cesarean section significantly escalates PAS risk, which is further compounded by the presence of placenta previa [5]. PAS is associated with serious maternal complications, including massive hemorrhage, large-volume transfusion requirements, and emergency peripartum hysterectomy [6]. Optimal management typically involves planned delivery at a tertiary care center by a multidisciplinary team [7]. Current guidelines from the American College of Obstetricians and Gynecologists recommend elective cesarean hysterectomy between 34^0^⁄_7_ and 35^6^⁄_7_ weeks of gestation in clinically stable patients [8,9]. However, early delivery and the surgical complexity inherent to PAS management may adversely affect neonatal outcomes.

Although various surgical techniques and maternal outcomes in PAS have been extensively studied, neonatal managements and outcomes remain relatively underexplored [10]. While pregnancies complicated by PAS are often associated with maternal hemorrhagic shock, limited evidence describes the corresponding neonatal hemodynamic and respiratory outcomes. Neonates born to PAS-complicated pregnancies have an increased risk of preterm birth, low birth weight (BW), small for gestational age (GA), low Apgar scores, and adverse composite outcomes [6,11]. Prior studies have also reported a higher incidence of respiratory complications, including respiratory distress syndrome and the need for endotracheal intubation, even after adjustment for GA [12,13].

Neonates born to mothers with PAS are at risk for respiratory compromise due to several mechanisms. Although prematurity remains the primary contributor due to American College of Obstetricians and Gynecologists recommendation on timing of delivery, emergency cesarean delivery due to sudden maternal bleeding or hemodynamic instability may result in suboptimal perinatal transition [14,15]. In addition, exposure to general anesthesia and perioperative sedative agents can contribute to neonatal respiratory depression. Abnormal placental invasion and vascular remodeling in PAS may impair uteroplacental perfusion, increasing the risk of fetal growth restriction, which has been associated with altered lung development [14]. Furthermore, acute maternal hemorrhage may compromise fetal oxygen delivery, potentially affecting neonatal cardiopulmonary adaptation after birth [16].

Nevertheless, limited evidence exists regarding predictors of respiratory compromise (RC) in neonates born to pregnancies complicated by PAS. To address this gap in knowledge, we aimed to identify key clinical predictors of RC in neonates born to pregnancies complicated by PAS and to develop a predictive model for early risk stratification.

## 2. Materials and Methods

### 2.1. Study Design and Participants

In this retrospective prognostic cohort study, we included neonates born to pregnant women diagnosed with PAS at Songklanagarind Hospital between 1 January 2014, and 31 December 2024. Neonates with major congenital anomalies involving the neurological, cardiovascular, or pulmonary systems and those with lethal chromosomal abnormalities (e.g., trisomy 13 or trisomy 18) were excluded from the analysis. Ethical approval for this study was obtained from the Human Research Ethics Committee of the Prince of Songkla University (REC. 67-309-1-1; date of approval: 14 August 2024).

### 2.2. Definitions

PAS was defined according to the clinical diagnostic criteria established by the International Federation of Gynecology and Obstetrics [17]. Hypertensive disorders included gestational hypertension, preeclampsia, and preeclampsia with severe features, as defined by the American College of Obstetricians and Gynecologists [18]. Diabetes mellitus, including gestational and overt diabetes, was defined using the Carpenter and Coustan criteria [19]. Antenatal corticosteroid administration was defined as receipt of at least one dose of dexamethasone before delivery.

We defined RC as the need for respiratory support, including either invasive ventilation (endotracheal intubation with mechanical ventilation) or non-invasive modalities, such as nasal continuous positive airway pressure, nasal high-frequency oscillatory ventilation, nasal intermittent positive pressure ventilation, or heated humidified high-flow nasal cannula. Hypoglycemia was defined according to the American Academy of Pediatrics Committee on Fetus and Newborn guidelines [20] and referred to a point-of-care glucose level < 47 mg/dL within the first 24 h of life. Neonatal hypotension was defined as systolic or diastolic blood pressure values more than two standard deviations (SDs) below the mean during the first 24 h of life, based on reference ranges from the Philadelphia Neonatal Blood Pressure Study Group [21].

Pulmonary hypertension was defined based on the diagnostic criteria for persistent pulmonary hypertension of the newborn, characterized by respiratory distress, refractory hypoxemia, and differential cyanosis between preductal and postductal sites, with confirmation by echocardiography performed by a neonatologist or pediatric cardiologist [22]. Newborns who had a right ventricular systolic pressure ≥ 50% of the systemic systolic pressure on echocardiography but did not meet criteria for persistent pulmonary hypertension of the newborn were also considered to have pulmonary hypertension [23]. Inotrope support was recorded when at least one vasoactive agent was administered within the first 24 h. The vasoactive–inotropic score was calculated using the highest recorded values during the first 72 h period. The score was defined as: dopamine dose (μg/kg/min) plus dobutamine dose (μg/kg/min) plus 100 × epinephrine dose (μg/kg/min) plus 100 × norepinephrine dose (μg/kg/min) plus 10,000 × vasopressin dose (U/kg/min) plus 10 × milrinone dose (μg/kg/min) [24]. Neonatal anemia was defined as a hematocrit level < 45% within the first 24 h of life. Blood transfusion was recorded if administered within the first 72 h of life.

### 2.3. Statistical Analysis

Data analysis was performed using the R software (version 4.4.2; Vienna, Austria). The demographic and clinical characteristics of mothers and neonates were compared between the RC and non-RC groups. All variables included in the final prediction model had complete data. A complete-case analysis approach was therefore applied, and no imputation was performed. Categorical variables are presented as frequencies and percentages and were analyzed using either the chi-square test or Fisher’s exact test, depending on the distribution of expected values. Continuous variables are reported as means ± SDs or medians with interquartile ranges (IQRs), depending on the data distribution. Comparisons between two groups were performed using Student’s *t*-test or Wilcoxon rank-sum test, as appropriate. The Kruskal–Wallis test was used for comparisons involving more than two groups. A two-sided *p* < 0.05 was considered statistically significant.

Univariable and multivariable analyses were performed. Independent variables with a *p* < 0.2 in the univariable analysis were included in a backward-stepwise multiple logistic regression, leading to the development of a clinical prediction scoring system. Notably, some predictors were incorporated into the scoring system despite not reaching statistical significance in the multivariable analysis because of their demonstrated association with adverse neonatal respiratory outcomes in previous studies and their established clinical relevance, as supported by the existing literature. To ensure model stability, multicollinearity among predictors was assessed using variance inflation factor scores. The areas under the receiver operating characteristic curve and calibration plot were calculated to evaluate model performance. The reliability was evaluated using sensitivity, specificity, predictive values, and likelihood ratios. Internal validation was conducted using bootstrap re-sampling with 5000 iterations to determine the robustness of the coefficient estimates.

## 3. Results

In total, 240 neonates were born to mothers diagnosed with PAS between 1 January 2014, and 31 December 2024. After excluding 3 neonates (major congenital anomalies [*n* = 2] or lethal chromosomal abnormalities [*n* = 1]), the final analysis included 237 neonates. Among these, 112 (47.3%) neonates developed RC **(**Table 1).

The mean maternal age was 35.0 ± 4.7 years, and the median body mass index (BMI) was 25.9 (23.1–28.8) kg/m^2^. Gestational diabetes and hypertensive disorders were reported in 13.9% and 5.5% of mothers, respectively. Vaginal bleeding within 24 h before delivery occurred in 31.6% of cases, and the median estimated blood loss was 2000 (100–4000) mL. Placenta types were distributed as follows: accreta in 24.9%, increta in 47.7%, and percreta in 27.4%. Nearly half of the mothers (49.4%) received at least one dose of antenatal corticosteroids, and 47.3% underwent emergency cesarean delivery. Maternal factors significantly associated with RC included vaginal bleeding within 24 h before delivery, greater estimated blood loss, placenta percreta, absence of antenatal corticosteroid administration, and emergency cesarean delivery (Table 1). No significant differences were observed between groups with respect to maternal age, BMI, diabetes mellitus, hypertensive disorders, number of prior cesarean deliveries, gravidity, parity, neonatal sex, or neonatal mortality.

The median GA was 35 (34–36) weeks, and the mean BW was 2555 ± 520 g. The median length of hospital stay was 6 (5–11) days. Hypovolemic shock occurred in 16% of neonates, all of whom required inotropic support; the median vasoactive–inotropic score was 3.2 (1.8–4.5).

Compared with neonates in the non-RC group, those in the RC group had significantly lower GA and BW, as well as a higher incidence of Apgar scores < 7 at 1 and 5 min and hypoglycemia. Respiratory complications, including pulmonary hypertension, respiratory distress syndrome, and transient tachypnea of the newborn, were also significantly more frequent in neonates with RC. In addition, RC was associated with higher rates of neonatal intensive care unit admission, hypovolemic shock, inotropic support, anemia, blood transfusion, and higher vasoactive–inotropic scores, along with longer hospital stays and lower hematocrit levels (Table 2).

In the univariate analysis, vaginal bleeding within 24 h before delivery, estimated blood loss, placenta percreta, absence of antenatal corticosteroid administration, emergency cesarean delivery, GA < 37 weeks, BW < 2500 g, male sex, and Apgar scores ≤ 7 at 1 and 5 min were identified as associated variables (Table 3). Multivariable logistic regression identified the following independent predictors of RC: vaginal bleeding within 24 h before delivery (adjusted odds ratio [aOR]: 2.09, 95% confidence interval [CI]: 1.04–4.24, *p* = 0.04), GA of 29–31 weeks (aOR: 12.02, 95% CI: 1.98–108.10, *p* = 0.01), GA of 32–36 weeks (aOR: 3.07, 95% CI: 1.08–10.23, *p* = 0.047), BW < 2500 g (aOR: 2.14, 95% CI: 1.12–4.15, *p* = 0.02), and male sex (aOR: 2.05, 95% CI: 1.10–3.86, *p* = 0.02) (Table 3).

A clinical prediction score for RC was developed based on significant independent predictors identified in the multivariable logistic regression analysis (Table 3). Notably, given their clinical relevance and potential additive value, placenta type and absence of antenatal corticosteroid administration, which did not retain their significant association with RC in the multivariable analysis, were incorporated into the final predictive scoring system [11,25]. Each variable was assigned weighted points by regression coefficient as follows: (1) maternal vaginal bleeding within 24 h before delivery: 2 points; (2) placenta type: increta: 1 point; percreta: 2 points; (3) absent antenatal corticosteroid use: 1 point; (4) GA: 29–31 weeks: 5 points; 32–36 weeks: 3 points; (5) BW < 2500 g: 2 points; and (6) male sex: 2 points. Multicollinearity was assessed using the generalized variance inflation factor. The adjusted generalized variance inflation factor for all variables are as follows: (1) maternal vaginal bleeding within 24 h before delivery: 1.08; (2) placenta type: 1.03; (3) absent antenatal corticosteroid use: 1.095; (4) GA: 1.01; (5) BW < 2500 g: 1.08; and (6) male sex: 1.07, indicating no evidence of problematic multicollinearity.

**Figure 1:**
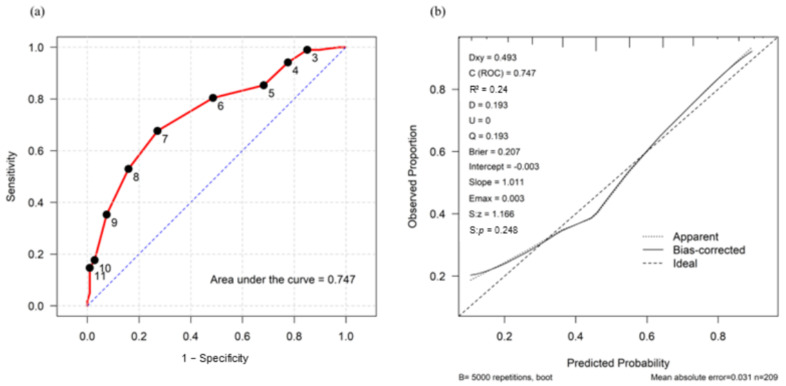
(**a**) Receiver operating characteristic curve of the clinical prediction model for neonatal respiratory compromise. The area under the curve is 0.75 (95% confidence interval: 0.68—0.81). At a cutoff score of 7, the model achieves optimal discrimination performance, yielding a sensitivity of 67.6% and specificity of 72.9%. (**b**) Calibration plot of the predictive model for neonatal respiratory compromise.

The area under the receiver operating characteristic curve (0.75) demonstrated good discrimination (Figure 1). An optimal cutoff score of 7 was identified, yielding a sensitivity of 67.6%, specificity of 72.9%, positive predictive value of 70.4%, and negative predictive value of 70.3% (Table 4). Model calibration was assessed using a bias-corrected calibration plot derived from 5000 bootstrap iterations, which showed a close agreement between predicted and observed outcomes (Figure 1). The model achieved a Brier score of 0.207, calibration slope of 1.011, and Emax of 0.003, indicating good overall performance and calibration.

## 4. Discussion

In this study, we identified the key clinical predictors of neonatal RC in PAS-complicated pregnancies and based on the identified predictors, introduced a practical prediction score for early risk assessment. RC may represent the earliest clinically threshold at which respiratory compromise necessitates intervention and NICU-level resources. In PAS deliveries, neonatal teams are frequently notified before birth and must anticipate monitoring needs and resource allocation prior to knowing the infant’s postnatal trajectory. Therefore, this model is designed as a prognostic tool for early risk stratification within a PAS population. The predictors including the scoring were maternal vaginal bleeding within 24 h before delivery, placenta type, absence of antenatal corticosteroid use, GA, BW < 2500 g, and male sex. This score demonstrated a good discrimination, and the optimal cutoff score was 7.

PAS has become an increasingly recognized obstetric condition associated with substantial maternal and neonatal morbidity. A large retrospective cohort study demonstrated that PAS-complicated pregnancy is associated with a higher incidence of adverse neonatal outcomes compared with pregnancy not complicated with PAS [11]. In our cohort, nearly half of the neonates born to PAS-complicated pregnancies (47.3%) experienced RC, which is consistent with findings from a previous study reporting increased rates of respiratory interventions, including endotracheal intubation and use of high-flow nasal cannula, among PAS-affected neonates [13]. Compared with neonates in the non-RC group, those in the RC group exhibited significantly higher rates of adverse outcomes, including lower GA and BW; higher frequencies of Apgar scores ≤ 7 at both 1 and 5 min; hypoglycemia; respiratory diseases; neonatal intensive care unit admission; prolonged hospitalization; hypotension; need for inotropic support; lower hematocrit levels; anemia; blood transfusion requirements; and elevated vasoactive–inotropic scores. These findings suggest that PAS-complicated pregnancies associated with increased neonatal morbidity and physiologic instability, predisposing them to both respiratory and multisystem instability.

The observed lower GA and BW are, in part, consistent with current clinical recommendations for elective cesarean hysterectomy between 34^0^⁄_7_ and 35^6^⁄_7_ weeks of gestation in clinically stable patients, a strategy intended to minimize maternal hemorrhagic risk [9]. Although this timing strategy benefits maternal outcomes, it inherently exposes neonates to complications related to physiologic immaturity. Lower GA and BW remain established risk factors for respiratory morbidity. In addition low Apgar scores at birth and hypoglycemia likely indicate perinatal stress further contributing to neonatal instability.

The increased burden of respiratory diseases, including transient tachypnea of the newborn, respiratory distress syndrome, and pulmonary hypertension, further underscores the vulnerability of neonates born to PAS-complicated pregnancies. Our findings are consistent with prior studies demonstrating significantly higher rates of respiratory distress syndrome and prolonged respiratory support among neonates from pregnancies complicated by abnormal placentation [12]. Moreover, the higher frequency of neonatal intensive care unit admission and longer hospital stays among neonates with RC highlight the need for intensive monitoring and specialized care. The high incidence of hypotension, inotropic support, anemia, transfusion requirements, and elevated vasoactive–inotropic scores reflects a broader pattern of cardiovascular and hematologic compromise, likely rooted in antenatal and perinatal hemodynamic instability associated with PAS. These neonatal findings parallel the maternal risk factors identified in our cohort, including vaginal bleeding within 24 h before delivery, greater estimated blood loss, and the presence of placenta percreta. Vaginal bleeding within 24 h before delivery did not uniformly indicate emergent cesarean delivery in our cohort. While some women were observed and subsequently underwent planned cesarean section, others required emergency delivery. Vaginal bleeding remained independently associated with neonatal respiratory compromise after adjustment for delivery urgency and other covariates, suggesting that its predictive value extends beyond simply reflecting emergency obstetric conditions. In our institutional practice, cesarean delivery for PAS is predominantly performed under general anesthesia due to the anticipated surgical complexity and hemorrhage risk. Therefore, anesthesia exposure showed minimal variability and is unlikely to explain the observed association. Male sex was also identified as an independent risk factor in previous population-based research, further supporting its inclusion in our model [26]. Additionally, the beneficial effect of antenatal corticosteroid administration in reducing the incidence and severity of respiratory distress has been well documented in clinical trials, reinforcing its role as a biologically plausible predictor in neonatal respiratory outcomes [27].

The predictive scoring model developed in this study provides a simple and practical tool for estimating the risk of RC among neonates born to PAS-complicated pregnancies. When applied using a cutoff score of 7, the post-test probability of 64% reflecting meaningful risk discrimination in this population. By identifying neonates at higher estimated risk, it may facilitate clinical assessment and multidisciplinary communication among obstetric, anesthesiology, and neonatal teams. The dominance of gestational age and birth weight in the model reflects fundamental determinants of neonatal physiologic prematurity. However, the model could be used for risk stratification within a PAS cohort, where respiratory outcomes are influenced by both maturity and peripartum stressors. Therefore, it should not be interpreted as evidence that PAS itself is the primary causal driver of respiratory compromise.

The predictive model demonstrated acceptable discriminative performance, with an area under the curve of 0.75 (95% CI: 0.68–0.81). Although sensitivity, specificity, and predictive values were modest, these findings suggest that the score is best suited as a risk stratification and risk stratification tool rather than as a primary screening or diagnostic instrument. The calibration plot showed close agreement between predicted and observed outcomes, and the low Brier score further supported the model’s reliability. Together, these results indicate that the model may serve as a practical approach for early risk stratification and structured clinical assessment in PAS-affected pregnancies.

This study has several notable strengths. It draws on a decade-long cohort from a tertiary referral center specializing in PAS, ensuring a clinically robust and relevant population. The use of multivariable modeling and internal validation through bootstrap re-sampling strengthens the credibility of the findings. Moreover, this study is among the first to propose a predictive scoring system specifically focused on neonatal RC in PAS-complicated pregnancies, addressing an important gap in the literature.

Some limitations should be acknowledged. First, the single-center design may limit the generalizability of the findings. Second, misclassification of exposure is possible because placenta type was primarily determined using prenatal ultrasonography, which may occasionally differ from pathological findings. Third, clinical practices evolved over the 11-year study period, potentially affecting comparability across years, particularly following the introduction of the multidisciplinary care team in 2016 [28]. Finally, although the prediction model demonstrated moderate discriminative performance, with an AUC of 0.75 and modest sensitivity and specificity, it should not be used as a standalone clinical decision tool. External validation in diverse clinical settings is required to confirm its broader applicability and reliability.

## 5. Conclusions

In conclusion, we identified the key clinical predictors of neonatal RC in PAS-complicated pregnancies and introduced a practical prediction score for early risk assessment. A score > 7 may help clinicians identify neonates at a higher estimated risk of RC. This model may assist in anticipatory clinical assessment and multidisciplinary communication within PAS care settings. Future research should focus on prospective validation and integration of this model into standardized PAS care pathways to optimize neonatal outcomes.

## Figures and Tables

**Table 1:** Maternal characteristics between the neonatal respiratory compromised (RC) and non-RC groups.

Maternal Characteristics	RC Group (*n* = 112)	Non-RC Groups (*n* = 125)	*p*-Value
Age, years ^a^	34.7 ± 4.7	35.3 ± 4.7	0.32
Body mass index, kg/m^2 b^	26.5 (23.7–29.5)	25.2 (22.8–28.0)	0.07
Body mass index in groups			0.19
≤18.5 kg/m^2^	3 (2.9)	2 (1.9)	
18.6–24.9 kg/m^2^	35 (34.3)	50 (46.3)	
≥25 kg/m^2^	64 (62.7)	56 (51.9)	
Diabetes mellitus	17 (15.2)	16 (12.8)	0.73
Hypertensive disorders	8 (7.1)	5 (4.0)	0.44
Number of previous cesarean deliveries, times ^b^	1 (1–2)	1 (1–2)	0.29
Gravidity ^b^	3 (3–4)	3 (2–4)	0.07
Gravidity in groups			0.15
1–2	25 (22.3)	42 (33.6)	
3–4	70 (62.5)	68 (54.4)	
≥5	17 (15.2)	15 (12)	
Placenta type at initial diagnosis			0.03
Accreta	25 (22.3)	34 (27.2)	
Increta	47 (42.0)	66 (52.8)	
Percreta	40 (35.7)	25 (20.0)	
Absence of antenatal corticosteroid use	46 (41.1)	74 (59.2)	0.01
Emergent cesarean delivery	63 (56.2)	49 (39.2)	0.01
Vaginal bleeding within 24 h before delivery	48 (42.9)	27 (21.6)	<0.001
Volume of blood loss, mL ^b^	2450 (1400–5000)	1800 (1000–3350)	0.01

Data are presented as numbers (%) unless indicated otherwise. ^a^ Mean ± standard deviation or ^b^ median (interquartile range).

**Table 2:** Neonatal characteristics and outcomes between the neonatal respiratory compromised (RC) and non-RC groups.

Neonatal Characteristics	RC Group (*n* = 112)	Non-RC Group (*n* = 125)	*p*-Value
Gestational age, weeks ^a^	35 (33–36)	36 (35–37)	<0.001
Gestational age in groups			<0.001
29–31 weeks	22 (19.6)	2 (1.6)	
32–36 weeks	85 (75.9)	98 (78.4)	
≥37 weeks	5 (4.5)	25 (20.0)	
Birth weight, g ^b^	2359 ± 511	2730 ± 464	<0.001
Birth weight in groups			<0.001
<2500 g	66 (58.9)	35 (28.0)	
≥2500 g	46 (41.1)	90 (72.0)	
Male	66 (58.9)	59 (47.2)	0.09
APGAR score at 1 min ^a^	6 (5–7)	8 (7–8)	<0.001
APGAR score at 5 min ^a^	8 (7–9)	9 (9–9)	<0.001
APGAR score at 1 min ≤ 7	22 (19.6)	1 (0.8)	<0.001
APGAR score at 5 min ≤ 7	24 (21.4)	3 (2.4)	<0.001
Hypoglycemia	8 (7.1)	2 (1.6)	0.05
Respiratory complications			0.03
Transient tachypnea of the newborn	52 (46.4)	5 (4.0)	<0.001
Respiratory distress syndrome	35 (31.2)	1 (0.8)	<0.001
Pulmonary hypertension	15 (13.4)	0 (0)	<0.001
**Neonatal Outcomes**	**RC Group (*n* = 112)**	**Non-RC Group (*n* = 125)**	***p*** **-Value**
Neonatal death	3 (2.7)	0 (0)	0.10
Neonatal intensive care unit admission	110 (98.2)	11 (8.8)	<0.001
Length of hospital stay, day ^a^	10 (6–15)	5 (4–7)	<0.001
Hypovolemic shock	37 (33.0)	1 (0.8)	<0.001
Inotrope support	37 (33.0)	1 (0.8)	<0.001
Vasoactive-inotropic score ^a^	6.6 (3.9–9.3)	0.1 (0–0.4)	<0.001
Hematocrit, % ^a^	46 (42–49)	50 (46–56)	<0.001
Anemia, Hct < 45%	46 (41.1)	20 (19.8)	0.001
Blood transfusion	13 (11.8)	0 (0)	<0.001

Data are presented as numbers (%) unless indicated otherwise. ^a^ Median (interquartile range) or ^b^ mean ± standard deviation.

**Table 3:** Maternal and neonatal factors associated with the neonatal respiratory compromised group.

Risk Factors	Univariable Analysis		Multivariable Analysis	
	Odds Ratio (95% CI)	*p*-Value	Odds Ratio (95% CI)	*p*-Value
Body mass index in groups (reference: ≤18.5 kg/m^2^)	1.00		-	-
18.6–24.9 kg/m^2^	0.47 (0.06–2.95)	0.42		
≥25 kg/m^2^	0.78 (0.10–4.84)	0.79		
Gravidity in groups (reference: 1–2)	1.00		-	-
3–4	1.89 (1.00–3.64)	0.06		
≥5	2.29 (0.92–5.85)	0.08		
Placenta type at initial diagnosis (reference: Accreta)	1.00		1.00	0.20
Increta	1.14 (0.57–2.28)	0.70	1.11 (0.51–2.42)	0.79
Percreta	2.55 (1.17–5.58)	0.02	1.99 (0.84–4.79)	0.12
Absent antenatal corticosteroid use	0.49 (0.28–0.85)	0.01	1.09 (0.57–2.11)	0.80
Emergency cesarean delivery	0.53 (0.30–0.92)	0.02	0.79 (0.40–1.53)	0.48
Vaginal bleeding within 24 h before delivery	2.92 (1.56–5.47)	<0.001	2.09 (1.04–4.24)	0.04
Maternal blood loss (reference: <2000 mL)	1.77 (1.02–3.06)	0.04	1.35 (0.70–2.61)	0.37
Gestational age in groups (reference: ≥37 weeks)	1.00		1.00	0.20
29–31 weeks	37.4 (7.72–293.17)	<0.001	12.02 (1.98–108.10747)	0.01
32–36 weeks	4.24 (1.65–7.21)	0.005	3.07 (1.08–10.23)	0.047
Birth weight < 2500 g	3.36 (1.90–6.03)	<0.001	2.14 (1.12–4.15)	0.02
Male sex	1.83 (1.06–3.18)	0.03	2.05 (1.10–3.86)	0.02
Apgar score at 1 min ≤ 7	8.91 (4.75–16.74)	<0.001	-	-
Apgar score at 5 min ≤ 7	29.15 (5.84–108.03)	<0.001	-	-
Hypoglycemia	4.47 (0.93–21.57)	0.06	-	-

CI: confidence interval.

**Table 4:** Prediction score and reliability for neonatal respiratory compromise.

Total Prediction Score	Sensitivity, % (95% CI)	Specificity, % (95% CI)	Positive Predictive Value, % (95% CI)	Negative Predictive Value, % (95% CI)	Probability, %
3	99.0 (94.7–100)	15.0 (8.8–23.1)	52.6 (45.3–59.8)	94.1 (71.3–99.9)	27.3
4	94.1 (87.6–97.8)	22.4 (14.9–31.5)	53.6 (46.0–61.1)	80.0 (61.4–92.3)	35.7
5	85.3 (76.9–91.5)	31.8 (23.1–41.5)	54.4 (46.3–62.3)	69.4 (54.6–81.7)	45.1
6	80.4 (71.0–87.6)	51.4 (41.5–61.2)	61.2 (52.4–65.5)	73.0 (61.9–82.5)	54.8
7	67.6 (57.7–76.6)	72.9 (63.4–81.0)	70.4 (60.3–79.2)	70.3 (60.9–78.6)	64.2
8	52.9 (42.8–62.9)	84.1 (75.8–90.5)	76.1 (64.5–85.4)	65.2 (56.6–73.1)	72.6
9	35.3 (26.1–45.4)	92.5 (85.8–96.7)	81.8 (67.3–91.8)	60.0 (52.1–67.5)	79.6
10	17.6 (10.8–26.4)	97.2 (92.0–99.4)	85.7 (63.7–97.0)	55.3 (47.9–62.6)	85.2
11	14.7 (8.5–23.1)	99.1 (94.9–100)	93.8 (89.8–99.8)	54.9 (47.6–62.1)	89.5
12	4.9 (1.6–11.1)	99.1 (94.9–100)	83.3 (35.9–99.6)	52.2 (45.1–59.3)	92.7

The total prediction score and its corresponding sensitivity and specificity for predicting neonatal respiratory compromise are shown. Reliability estimates are presented with 95% confidence intervals.

## Data Availability

The raw data supporting the conclusions of this study will be provided by the authors upon request. The data are not publicly available due to privacy concerns.

## Abbreviations

The following abbreviations are used in this manuscript:

aOR	Adjusted odds ratio
BW	Birth weight
CI	Confidence interval
GA	Gestational age
PAS	Placenta accreta spectrum
RC	Respiratory compromise
